# Functional diversification process of opsin genes for teleost visual and pineal photoreceptions

**DOI:** 10.1007/s00018-024-05461-3

**Published:** 2024-10-08

**Authors:** Chihiro Fujiyabu, Fuki Gyoja, Keita Sato, Emi Kawano-Yamashita, Hideyo Ohuchi, Takehiro G. Kusakabe, Takahiro Yamashita

**Affiliations:** 1 Department of Biophysics, Graduate School of Science, Kyoto University, Kyoto, 606-8502 Japan; 2 Institute for Integrative Neurobiology and Department of Biology, Graduate School of Natural Science, Konan University, Hyogo, 658-8501 Japan; 3 Department of Cytology and Histology, Faculty of Medicine, Dentistry and Pharmaceutical Sciences, Okayama University, Okayama, 700-8558 Japan; 4 Department of Chemistry, Biology and Environmental Science, Faculty of Science, Nara Women’s University, Nara, 630-8506 Japan

**Keywords:** Opsin, Rhodopsin, Pinopsin, Retroduplication, Retina, Pineal gland

## Abstract

Most vertebrates have a *rhodopsin* gene with a five-exon structure for visual photoreception. By contrast, teleost fishes have an intron-less *rhodopsin* gene for visual photoreception and an intron-containing *rhodopsin* (*exo-rhodopsin*) gene for pineal photoreception. Here, our analysis of non-teleost and teleost fishes in various lineages of the Actinopterygii reveals that retroduplication after branching of the Polypteriformes produced the intron-less *rhodopsin* gene for visual photoreception, which converted the parental intron-containing *rhodopsin* gene into a pineal opsin in the common ancestor of the Teleostei. Additional analysis of a pineal opsin, pinopsin, shows that the *pinopsin* gene functions as a green-sensitive opsin together with the intron-containing *rhodopsin* gene for pineal photoreception in tarpon as an evolutionary intermediate state but is missing in other teleost fishes, probably because of the redundancy with the intron-containing *rhodopsin* gene. We propose an evolutionary scenario where unique retroduplication caused a “domino effect” on the functional diversification of teleost visual and pineal opsin genes.

**Supplementary Information:**

The online version contains supplementary material available at 10.1007/s00018-024-05461-3.

## Introduction

Most animals have photoreceptive organs and cells to capture various kinds of light information from the outer environment. Opsins are universal photoreceptor proteins which work in the photoreceptive organs for visual and non-visual functions in animals [1–3]. Recent accumulation of genomic information has revealed that animals have various opsin genes which are classified into several groups based on their amino acid sequences. Opsins share amino acid sequence homology and structural elements including seven transmembrane domains and a chromophore, retinal. Thus, opsin genes are considered to have been diversified by gene duplications and mutations from an ancestral opsin gene, which has led to the different molecular properties and expression patterns of the proteins that they encode for the visual and non-visual photoreceptions.

Among these opsins, vertebrate rhodopsin is the best-studied opsin responsible for visual photoreception [4]. Vertebrate rhodopsin works in rod cells of the retina and binds 11-*cis* retinal in the dark. After visible light reception of rhodopsin, the isomerization of the retinal to all-*trans* form induces the formation of the active state, meta II intermediate, whose absorption maximum (λmax) lies in the UV region. Meta II activates G protein transducin, which eventually evokes the hyperpolarization of rod cells. The search for the *rhodopsin* gene in vertebrate genomes has shown that many vertebrates have a single *rhodopsin* gene which has a five exons/four introns structure [5]. On the other hand, teleost fishes exceptionally have two types of *rhodopsin* genes, one of which has the five exons/four introns structure and the other of which has a single exon (intron-less) structure [6–8]. This intron-less *rhodopsin* gene is considered to have been acquired by RNA-based gene duplication (retroduplication, also known as retrotransposition) from the parental intron-containing *rhodopsin* gene [9]. It has been reported that these two *rhodopsin* genes exhibit different expression patterns in the Teleostei. In general, the intron-less *rhodopsin* gene works in rod cells of the retina in teleost fishes [6]. By contrast, the intron-containing *rhodopsin* gene exclusively works in the pineal gland of zebrafish, which led to the gene being named *exo-rhodopsin* (extra-ocular *rhodopsin*) [10]. In addition, meta II of the rhodopsin protein encoded by the intron-less *rhodopsin* gene has a different property from meta II of the exo-rhodopsin protein encoded by the intron-containing *rhodopsin* gene. That is, meta II of exo-rhodopsin protein in the pineal gland decays faster than meta II of the rhodopsin protein in rod cells of the retina [11, 12]. This can be adaptive for pineal photoreception under bright conditions by facilitating bleach recovery of photo-pigment. These features of two *rhodopsin* genes provide an evolutionary model for the emergence of the intron-less *rhodopsin* gene in the Teleostei. In the Actinopterygii, an intron-less *rhodopsin* gene was formed by retroduplication from the intron-containing *rhodopsin* gene. This intron-less *rhodopsin* gene was utilized in the retina, whereas the parental intron-containing *rhodopsin* gene changed its distribution pattern to abundant expression in the pineal gland accompanied by the change of the molecular property of the protein that it encoded.

In previous studies, to unveil the molecular origin of the teleost-specific intron-less *rhodopsin* gene, we analyzed the *rhodopsin* genes of non-teleost fishes in the Actinopterygii [13, 14]. Our analysis suggested that the intron-less *rhodopsin* gene emerged by retroduplication after branching of the Polypteriformes, and the parental intron-containing *rhodopsin* gene changed the distribution pattern of its mRNA and the molecular property of the protein that it encoded after branching of the Holostei. In parallel, we also analyzed another pineal opsin gene, *pinopsin*. The *pinopsin* gene was originally identified as a gene that functions in the chicken pineal gland and forms a blue-sensitive photo-pigment [15]. This *pinopsin* gene is most closely related to vertebrate visual opsin genes, including the *rhodopsin* gene, in the phylogenetic tree, which results in sharing of molecular properties between rhodopsin protein and pinopsin protein, such as the formation of meta II as an active state after photoreception and the coupling with transducin [16, 17]. Recent analysis in several vertebrates showed that the *pinopsin* gene is expressed in the pineal gland and its related organs of birds, reptiles, amphibians and non-teleost fishes [13, 15, 17–19]. By contrast, the *pinopsin* gene has not been identified in the genomes of the Teleostei [20]. Therefore, because of the similar molecular properties between rhodopsin and pinopsin proteins, we speculated that several teleost fishes utilize the intron-containing *rhodopsin* gene for pineal photoreception instead of the *pinopsin* gene. However, limited genomic information of teleost fishes in the Osteoglossomorpha and Elopomorpha, which branched before the diversification in the Clupeocephala, prevented the detailed analysis of the functional diversification of the two *rhodopsin* genes and *pinopsin* gene in the early evolutionary process of the Teleostei.

In this study, based on recent advances in genome analysis in the Osteoglossomorpha and Elopomorpha, we analyzed the *rhodopsin* and *pinopsin* genes of teleost fishes in these lineages. We identified intron-containing and intron-less *rhodopsin* genes in several fishes of the Osteoglossomorpha and Elopomorpha and revealed the abundant expressions of the intron-less *rhodopsin* gene in the retina and of the intron-containing *rhodopsin* gene in the pineal gland. This expression pattern of the intron-containing *rhodopsin* gene was quite different from that in non-teleost fishes such as spotted gar (*Lepisosteus oculatus*) and gray bichir (*Polypterus senegalus*) [13]. Moreover, surprisingly, we identified a *pinopsin* gene in the genome of Atlantic tarpon (*Megalops atlanticus*) in the Elopomorpha. This *pinopsin* gene encoded a green-sensitive opsin protein and was expressed in the pineal gland together with the intron-containing *rhodopsin* gene. Thus, Atlantic tarpon remains in an evolutionary intermediate state between non-teleost fishes whose pineal gland expresses the *pinopsin* gene and teleost fishes whose pineal gland expresses the intron-containing *rhodopsin* gene. These analyses support an evolutionary scenario in which unique retroduplication would have triggered sequential diversification of the visual and pineal opsin genes in the Actinopterygii.

## Materials and methods

### Animals and ethics statement

Australian bonytongues (*Scleropages jardinii,* body length: ~ 10 cm) and Atlantic tarpons (*M. atlanticus,* 10 ~ 20 cm) were purchased from local pet shops. Japanese eels (*Anguilla japonica,* ~ 30 cm) were purchased from a local fish shop. The use of animals in these experiments was in accordance with guidelines established by the Japan Ministry of Education, Culture, Sports, Science and Technology. The experiments in this report were approved by the Animal Care and Use Committee of Kyoto University (permit number: 202304 and 202404).

### Isolation of cDNA encoding opsin

To isolate the clones of Japanese eel intron-less *rhodopsin* genes (*fw-rho* and *ds-rho*) and intron-containing *rhodopsin* gene, we searched them in genomic databases and identified their full-length ORF sequences. The clones of Japanese eel *fw-rho* (accession no. LC464071.1), *ds-rho* (LC464070.1) and intron-containing *rhodopsin* gene (LC464064.1) were isolated by PCR from 1st strand cDNA from the eyes or brain as described in our previous studies [14]. To isolate Australian bonytongue intron-less *rhodopsin* gene, we first obtained the sequence including the full-length ORF (the single exon) by PCR from the genomic DNA based on the homology to the sequence of Asian arowana (*Scleropages formosus*) orthologue (XM_018734111.1). Primer sequences were as follows: 5′-GGACTGACGGCGAGCGGCAG-3′ (forward) and 5′-CCGAAAACGGGAGCCTTGTTC-3′ (reverse). After the identification of the start codon and the stop codon of the ORF, the full-length ORF of Australian bonytongue intron-less *rhodopsin* gene (LC818096) was obtained from eyes. To isolate the Australian bonytongue intron-containing *rhodopsin* gene, we first obtained the sequences of exon 1 (including the start codon of ORF) and exon 5 (including the stop codon of ORF) by PCR from the genomic DNA based on the homology to the sequence of the Asian arowana orthologue (XM_018762271.1). Primer sequences were as follows: 5′- CATAAGGCGGGAAACTGCAG-3′ (forward) and 5′- TGCTGGATGGTGACGTAGAG-3′ (reverse) for exon 1 and 5′- GTTTCGGAACTGCATGATCA-3′ (forward) and 5′- GATGGCTTTCCCAGGCGCTGGG-3′ (reverse) for exon 5. After the identification of the start and stop codons of ORF, the full-length ORF of Australian bonytongue intron-containing *rhodopsin* gene (LC818097) was obtained from the brain. To isolate Atlantic tarpon *rhodopsin* and *pinopsin* genes, we searched them in the genomic database (GCA_019176425.1) and identified their full-length ORF sequences. The clones of Atlantic tarpon intron-less *rhodopsin* gene (LC818093), intron-containing *rhodopsin* gene (LC818094), and *pinopsin* gene (LC818095) were obtained from eyes or brain by PCR.

### Preparation of recombinant proteins

The cDNAs encoding opsins were tagged with the epitope sequence of the anti-bovine rhodopsin monoclonal antibody Rho1D4 (ETSQVAPA) at the C-terminus and introduced into the mammalian expression vector pCAGGS [21]. Site-directed mutations were introduced using the seamless ligation cloning extract (SLiCE) method [22] or the In-Fusion cloning kit (Clontech). The plasmid DNA was transfected into HEK293S cells using the calcium phosphate method. The cells were collected 48 h after transfection and incubated with 40 μM 11-*cis* retinal at 4 °C for 24 h in the dark. The reconstituted photo-pigments were extracted from cell membranes with 1% dodecyl maltoside (DDM) in Buffer A (50 mM HEPES, 140 mM NaCl, pH 6.5). For purification, the extract was incubated with Rho1D4-conjugated agarose overnight and washed with 0.02% DDM in Buffer A. The purified photo-pigments were eluted with 0.02% DDM in Buffer A containing the synthetic peptide that corresponds to the C-terminus of bovine rhodopsin. All of the procedures were carried out on ice under dim red light.

### Spectroscopic measurements

UV/Vis absorption spectra were recorded using a spectrophotometer (UV2450 or UV2600, Shimadzu) and an optical cell (width, 2 mm; light path, 1 cm). The sample temperature was maintained using a temperature controller (RTE-210, NESLAB) at 0 ± 0.1 °C. The sample was irradiated with yellow light (> 500 nm) which was generated by a 1-kW tungsten halogen lamp (Master HILUX-HR, Rikagaku Seiki) and passed through an optical filter (Y-52, AGC Techno Glass). The decay of meta II of rhodopsin and exo-rhodopsin was measured by monitoring the intrinsic tryptophan fluorescence emission using a fluorescence spectrophotometer (RF5300, Shimadzu). 60 nM photo-pigments in Buffer A containing 0.02% DDM were irradiated for 10 s with yellow light (> 500 nm) which was generated by a 1-kW tungsten halogen lamp (Master HILUX-HR, Rikagaku Seiki) and passed through an optical filter (Y-52, AGC Techno Glass), and the change of the fluorescence emission at 340 nm induced by the retinal release at room temperature was observed. Experimental data were fitted by a single exponential function to calculate the decay rate of meta II.

### GTPγS binding assay

The activation of transducin by the pinopsin proteins was measured by GDP/GTPγS exchange of G protein using a radionucleotide filter-binding assay [16]. Trimeric transducin was purified from the bovine retina [23]. All of the assay procedures were carried out at 15 °C. The assay mixture consisted of 10 nM pinopsin protein purified after reconstitution with 11-*cis* retinal, 600 nM transducin protein, 1 μM [^35^S]GTPγS, 2 μM GDP, 50 mM HEPES, 140 mM NaCl, 5 mM MgCl_2_, 1 mM dithiothreitol (DTT), 0.01% DDM, pH 7.0. Pinopsin protein was mixed with transducin and was kept in the dark or irradiated for 30 s with yellow light (> 500 nm). After irradiation, the GDP/GTPγS exchange reaction was initiated by the addition of [^35^S]GTPγS solution to the mixture of pinopsin and transducin. After incubation for the selected time in the dark, an aliquot (20 μL) was transferred from the sample into 200 μL of stop solution (20 mM Tris/Cl, 100 mM NaCl, 25 mM MgCl_2_, 1 μM GTPγS and 2 μM GDP, pH 7.4), and the mixture was immediately filtered through a nitrocellulose membrane to trap [^35^S]GTPγS bound to transducin. The amount of bound [^35^S]GTPγS was quantitated by assaying the membrane with a liquid scintillation counter (Tri-Carb 2910 TR, PerkinElmer).

### Preparation of tissue sections

After the eyes and brains were dissected from Australian bonytongue, Japanese eel, or Atlantic tarpon, they were fixed overnight in PBS containing 4% or 6% PFA at 4 °C. The fixed tissues were soaked in PBS containing 20% sucrose overnight at 4 °C for cryoprotection, embedded in OCT compound (Tissue-Tek), and frozen at − 80 °C. Frozen tissues were sliced into 15- or 16-μm sections and affixed to glass slides. For the sagittal sections of the Atlantic tarpon brain, brain tissues including skin and cranium were fixed overnight in Bouin’s fixative at 4 °C. The fixed tissues were soaked in PBS containing 20% sucrose and 0.5 M EDTA at 4 °C for 48 h for cryoprotection and further decalcification. The tissues were subsequently washed with PBS containing 20% sucrose, embedded in OCT compound, and frozen at − 80 °C. Frozen tissues were sliced into 20-μm sections and affixed to glass slides. These samples were dried and stored at − 20 °C until use.

### Alcian blue staining

Some sagittal sections of Atlantic tarpon head were stained with Alcian Blue. After post-fixation in 95% ethanol for 1 min and removal of OCT compound with water, the sections were immersed in 3% (v/v) acetic acid for 3 min. They were then stained with Alcian Blue solution (pH 2.5; FUJIFILM Wako) for 10 min, followed by immersion in 3% (v/v) acetic acid for 1 min and in water for 1 min. Subsequently, the sections were counterstained with Nuclear Fast Red, washed in tap water, dehydrated twice in isopropanol for 2 min, and coverslipped with VectaMount Express Mounting Medium (Vector Laboratories).

### In situ hybridization

In situ hybridization was performed according to a previous study [14]. Digoxigenin-labeled riboprobes of opsin genes were synthesized from full-length cDNAs flanked by T7 and T3 promoter sequences which were inserted into pTA2 (TOYOBO). Tissue sections were sequentially immersed in 4% PFA in PBS for 15 min, 100% methanol for 30 min, PBS for 5 min, Proteinase K (0.5 μg/mL)/Tris–EDTA buffer (50 mM Tris–HCl, 5 mM EDTA, pH 7.6) for 15 min, 4% PFA in PBS for 15 min, dimethyl dicarbonate-treated water for 30 s, acetylation buffer (0.27% (v/v) acetic anhydride, 100 mM triethanolamine, pH 8.0) for over 30 min, PBS for 5 min, and hybridization buffer (750 mM NaCl, 75 mM sodium citrate, 0.4 mg/mL yeast RNA, 0.1 mg/mL heparin sodium, 1 × Denhardt’s solution, 0.1% (v/v) Tween, 0.1% (w/v) CHAPS, 5 mM EDTA, 70% (v/v) formamide) at 65 °C for 2 ~ 3 h. Digoxigenin-labelled riboprobes (final concentration: 0.17 μg/mL) diluted in hybridization buffer were then applied to tissue sections and incubated at 65 °C for approximately 40 h.

After hybridization, the tissue sections were washed in 1 × SSC buffer (150 mM NaCl, 15 mM sodium citrate, pH 7.0) containing 50% formamide at 65 °C for 15 ~ 30 min and 1 ~ 1.5 h, followed by washing in 0.2 × SSC buffer at 65 °C for 1 ~ 1.5 h and MABT (100 mM maleate, 150 mM NaCl, 0.1% Tween 20, pH 7.5) three times for 0.5 ~ 1 h each. After washing, tissue sections were soaked in blocking buffer (1% (w/v) bovine serum albumin, 10% (v/v) sheep normal serum, 0.08% (v/v) Triton-X100 in PBS) for 0.5 ~ 1.5 h and then incubated with alkaline phosphatase-conjugated anti-digoxigenin Fab fragment (1:2000 or 1:5000 (for the Atlantic tarpon eye sections) dilution; Roche) overnight at 4 °C.

Tissue sections were then rinsed three times for 30 min each with MABT and twice for 5 min each with AP reaction buffer (100 mM Tris–HCl, 50 mM MgCl_2_, 100 mM NaCl, and 0.1% Tween 20, pH 9.5). Color development was performed with 50 μg/mL nitro blue tetrazolium, 175 μg/mL 5-bromo-4-chloro-3-indolyl phosphate, 1 mM levamisole hydrochloride, and 5% (w/v) polyvinyl alcohol in AP buffer at 28 °C for 2 ~ 5 h. Tissue sections were immersed for 5 min in PBS containing 0.1% Triton X-100 and rinsed with water. After washing, tissue sections were counterstained with Nuclear Fast Red. Subsequently, the tissue sections were washed in tap water for 5 min, dehydrated twice in isopropanol for 2 min, and coverslipped with VectaMount Express Mounting Medium. All of the procedures were performed at room temperature unless otherwise noted.

## Results

### Synteny analysis of *rhodopsin* and *pinopsin* genes in the Actinopterygii

**Fig. 1:**
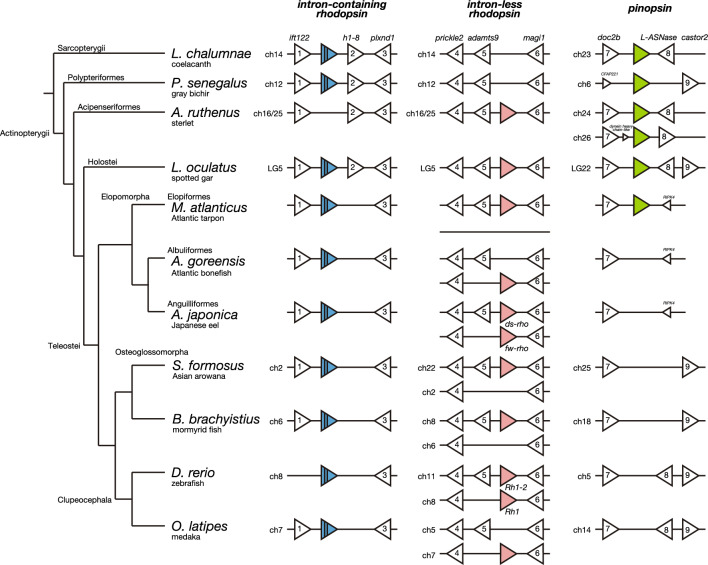
The synteny analysis of *rhodopsin* and *pinopsin* genes in the Actinopterygii. The synteny block of orthologous genes flanking the intron-containing *rhodopsin* gene (blue triangle with vertical lines), the intron-less *rhodopsin* gene (pink triangle), and the *pinopsin* gene (light green triangle) in actinopterygian species. The genes flanking the opsin loci are shown by white triangles. Gene names are indicated above the coelacanth (*Latimeria chalumnae*) genes, *intraflagellar transport 122* (*ift122*) (gene number 1 shown by white triangle), *H1.8 linker histone* (*h1-8*) (gene number 2), *plexin D1* (*plxnd1*) (gene number 3), *prickle planar cell polarity protein 2* (*prickle2*) (gene number 4), *ADAM metallopeptidase with thrombospondin type 1 motif 9* (*adamts9*) (gene number 5) and *membrane-associated guanylate kinase, WW and PDZ domain containing 1* (*magi1*) (gene number 6), *double C2 domain beta* (*doc2b*) (gene number 7), *L-asparaginase* (*L-ASNase*) (gene number 8), and *cytosolic arginine sensor for mTORC1 subunit 2* (*castor2*) (gene number 9). In Atlantic tarpon (*M. atlanticus*), regarding the two intron-less *rhodopsin* genes, the gene flanking *adamts9* is found in the conserved synteny block, whereas the genome region including the other gene is deleted. Detailed gene information is shown in Supplementary file 2

In a previous study, we isolated the *rhodopsin* genes from non-teleost fishes in the Actinopterygii [13]. In this study, we first searched for the *rhodopsin* genes in the genomes of teleost fishes in the Osteoglossomorpha and Elopomorpha and compared the syntenies in the Actinopterygii (Fig. 1 and Supplementary Data 1). An intron-containing *rhodopsin* gene, an orthologue of the tetrapod *rhodopsin* gene, is found in Asian arowana (*S. formosus*) and the mormyrid fish (*Brienomyrus brachyistius*) in the Osteoglossomorpha, and in Atlantic tarpon, Atlantic bonefish (*Albula goreensis*) and Japanese eel (*A. japonica*) in the Elopomorpha. The synteny block around this intron-containing *rhodopsin* gene in the Teleostei is generally conserved with that in gray bichir in the Polypteriformes and spotted gar in the Holostei. This *rhodopsin* gene is missing in the corresponding synteny block of sterlet (*Acipenser ruthenus*). Another *rhodopsin* gene, an intron-less *rhodopsin* gene, is found in sterlet and spotted gar, but not in gray bichir in the Polypteriformes, among non-teleost fishes in the Actinopterygii. Thus, it can be speculated that the intron-less *rhodopsin* gene emerged by retroduplication after branching of the Polypteriformes in the Actinopterygii, which is consistent with our previous molecular phylogenetic analysis of the *rhodopsin* genes found in non-teleost fishes [13]. This intron-less *rhodopsin* gene is found in the conserved synteny block of Asian arowana and the mormyrid fish in the Osteoglossomorpha, and in Atlantic tarpon, Atlantic bonefish, and Japanese eel in the Elopomorpha. Among these teleost fishes, Japanese eel has two intron-less *rhodopsin* genes, freshwater type (*fw-rho*) and deep-sea type (*ds-rho*), which are thought to have been duplicated by teleost-specific whole genome duplication [24]. Thus, it is supposed that other fishes lost one intron-less *rhodopsin* gene during the evolutionary process [7]. A detailed comparison of the syntenies of the two intron-less *rhodopsin* genes showed that Atlantic bonefish lost the gene flanking *adamts9*, whereas Asian arowana, the mormyrid fish and Atlantic tarpon lost the other gene. This suggests that a loss of an intron-less *rhodopsin* gene occurred independently and randomly in the Osteoglossomorpha and Elopomorpha lineages.

In previous studies, we identified a *pinopsin* gene from non-teleost fishes (spotted gar, Siberian sturgeon (*Acipenser baerii*) and gray bichir) and showed its abundant expression in the pineal gland [13, 17]. However, the *pinopsin* gene has not been identified in the genomes of the Teleostei [20]. In this study, we searched for the *pinopsin* gene in the genomes of the Osteoglossomorpha and Elopomorpha. We successfully identified a *pinopsin* gene in the genomes of two species, Atlantic tarpon and Indo-Pacific tarpon (*Megalops cyprinoides*), in the Elopiformes of the Elopomorpha. The synteny block around the *pinopsin* gene in Atlantic tarpon is generally conserved with that of non-teleost fishes. By contrast, the *pinopsin* gene is missing in the corresponding synteny block of other teleost fishes.

### Analysis of expression patterns of *rhodopsin* and *pinopsin* genes

**Fig. 2:**
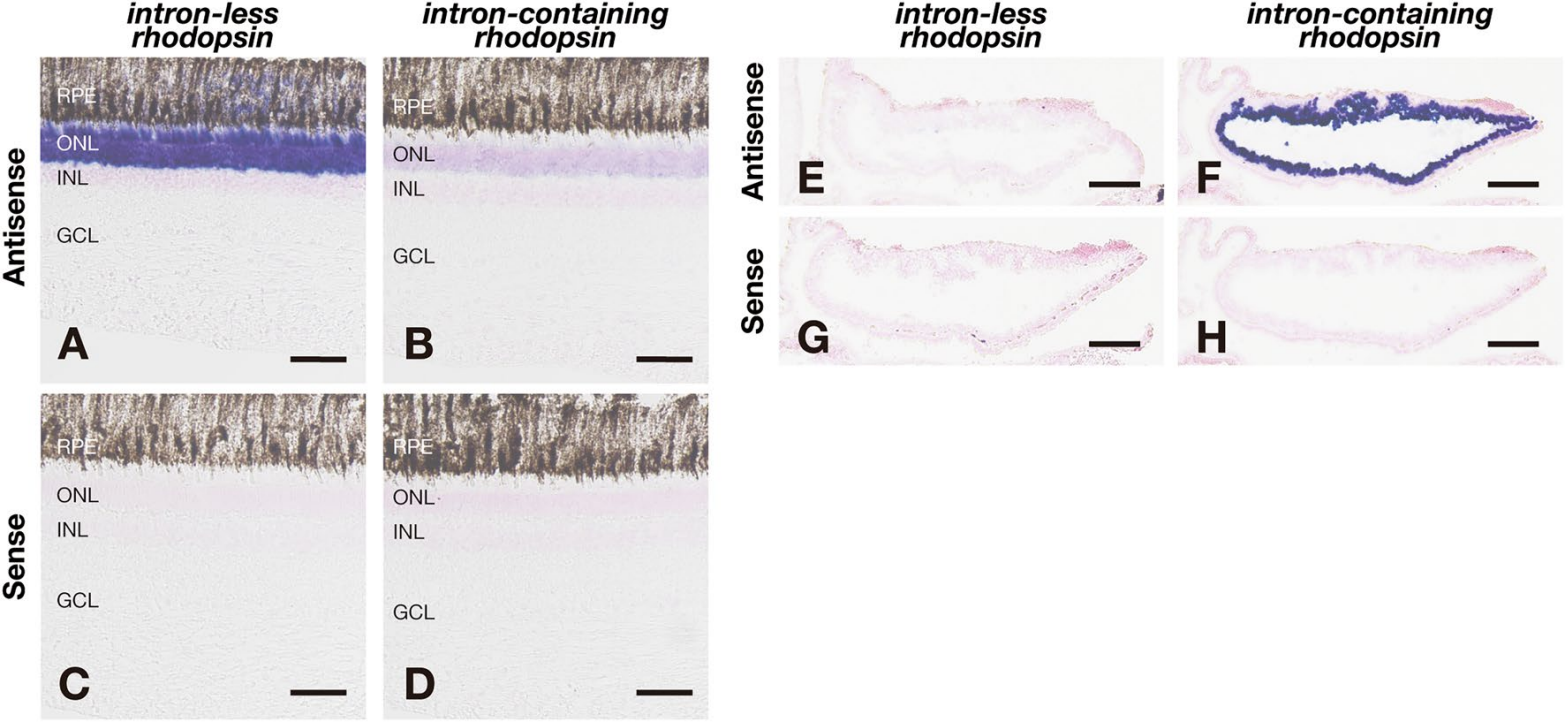
Distribution of mRNA of the intron-less and intron-containing *rhodopsin* genes in the retina and the pineal gland of Australian bonytongue. **A**-**D**, Distribution of the transcripts of Australian bonytongue intron-less *rhodopsin* gene (**A**, **C**) and intron-containing *rhodopsin* gene (**B**, **D**) in the retina. These sections were hybridized with antisense probes (**A**, **B**) or corresponding sense probes (**C**, **D**). Scale bar: 50 μm. Abbreviations: RPE, retinal pigment epithelium; ONL, outer nuclear layer; INL, inner nuclear layer; GCL, ganglion cell layer. **E**–**H**, Distribution of the transcripts of Australian bonytongue intron-less *rhodopsin* gene (**E**, **G**) and intron-containing *rhodopsin* gene (**F**, **H**) in the transverse sections of the pineal gland. These sections were hybridized with antisense probes (**E**, **F**) or corresponding sense probes (**G**, **H**). Scale bar: 100 μm

**Fig. 3:**
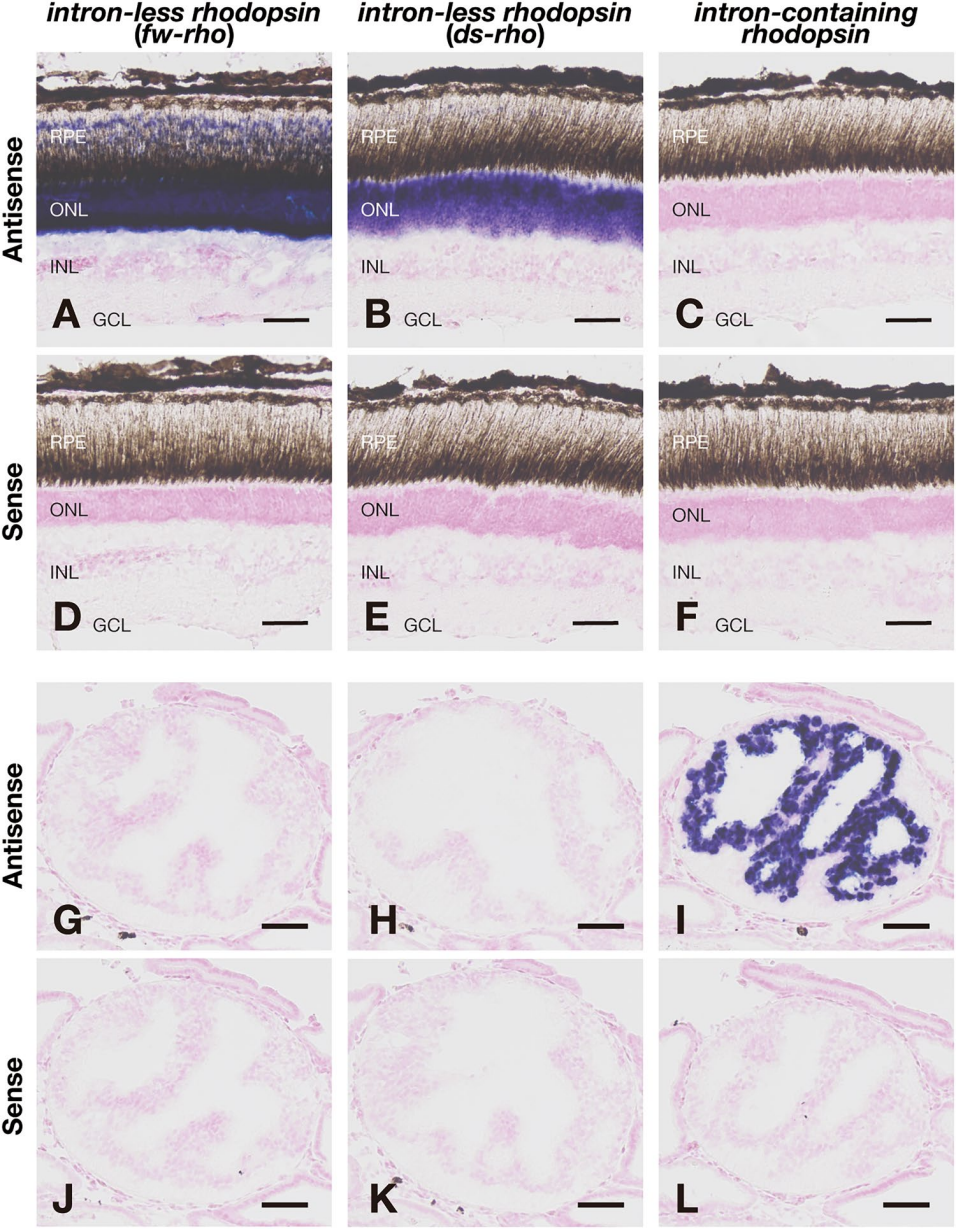
Distribution of mRNA of the intron-less and intron-containing *rhodopsin* genes in the retina and the pineal gland of Japanese eel. **A**–**F**, Distribution of the transcripts of Japanese eel intron-less *rhodopsin* genes, namely freshwater type *rhodopsin* gene (*fw-rho*) (**A**, **D**) and deep-sea type *rhodopsin* gene (*ds-rho*) (**B**, **E**), and intron-containing *rhodopsin* gene (**C**, **F**) in the retina. These sections were hybridized with antisense probes (**A**–**C**) or corresponding sense probes (**D**–**F**). Scale bar: 50 μm. Abbreviations: RPE, retinal pigment epithelium; ONL, outer nuclear layer; INL, inner nuclear layer; GCL, ganglion cell layer. **G**–**L**, Distribution of the transcripts of Japanese eel *fw-rho* gene (**G**, **J**), *ds-rho* gene (**H**, **K**), and intron-containing *rhodopsin* gene (**I**, **L**) in the transverse sections of the pineal gland. These sections were hybridized with antisense probes (**G**–**I**) or corresponding sense probes (**J**–**L**). Scale bar: 50 μm

**Fig. 4:**
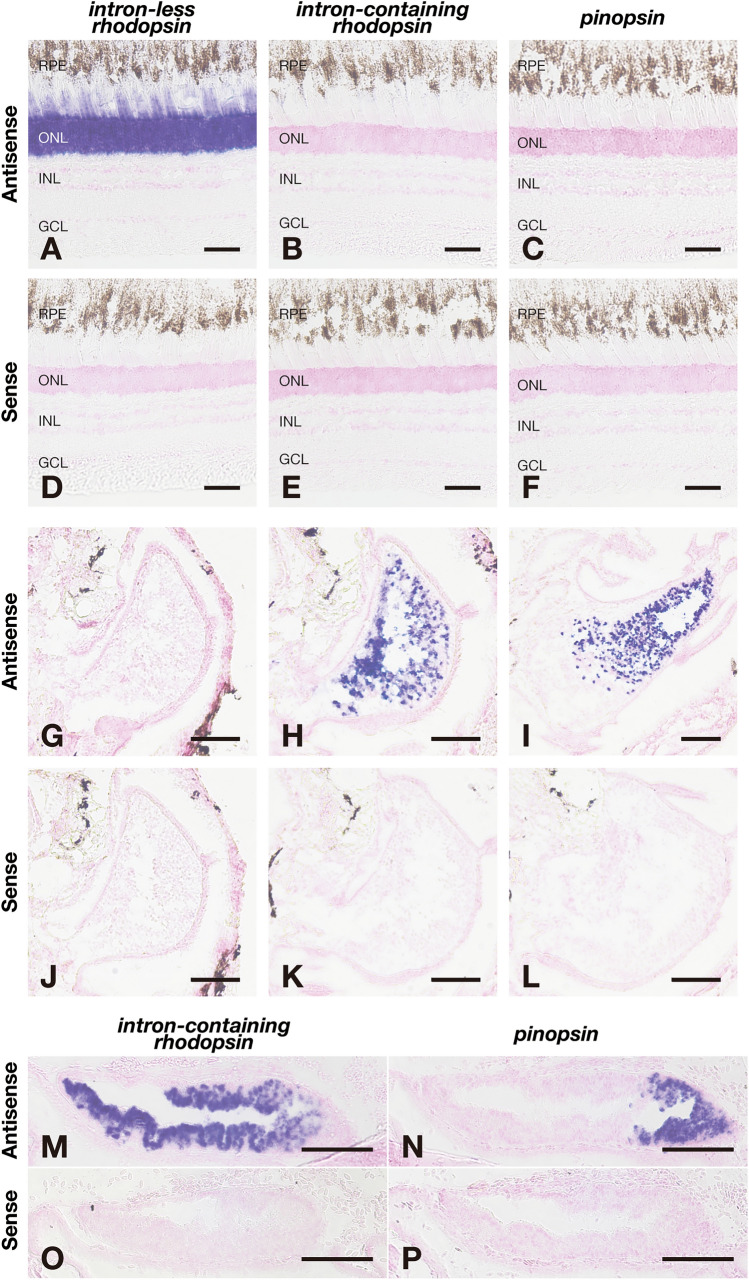
Distribution of mRNA of the intron-less and intron-containing *rhodopsin* genes and *pinopsin* gene in the retina and the pineal gland of Atlantic tarpon. **A**–**F**, Distribution of the transcripts of Atlantic tarpon intron-less *rhodopsin* gene (**A**, **D**), intron-containing *rhodopsin* gene (**B**, **E**), and *pinopsin* gene (**C**, **F**) in the retina. These sections were hybridized with antisense probes (**A**–**C**) or corresponding sense probes (**D**–**F**). Scale bar: 50 μm. Abbreviations: RPE, retinal pigment epithelium; ONL, outer nuclear layer; INL, inner nuclear layer; GCL, ganglion cell layer. **G**–**L**, Distribution of the transcripts of Atlantic tarpon intron-less *rhodopsin* gene (**G**, **J**), intron-containing *rhodopsin* gene (**H**, **K**), and *pinopsin* gene (**I**, **L**) in the transverse sections of the pineal gland. These sections were hybridized with antisense probes (**G**–**I**) or corresponding sense probes (**J**–**L**). Scale bar: 100 μm. **M**–**P**, Distribution of the transcripts of Atlantic tarpon intron-containing *rhodopsin* gene (**M**, **O**) and *pinopsin* gene (**N**, **P**) in the sagittal sections of the pineal gland. Rostral is to the left, and dorsal is up. These sections were hybridized with antisense probes (**M**, **N**) or corresponding sense probes (**O**, **P**). Scale bar: 100 μm

Analyses of *rhodopsin* genes in some teleost fishes showed that the intron-less *rhodopsin* gene is utilized in the retina, whereas the intron-containing *rhodopsin* gene is abundantly and exclusively expressed in the pineal gland [10, 25]. On the other hand, our previous study detected the abundant expression of both the intron-less and intron-containing *rhodopsin* genes in the retina, not in the pineal gland, of spotted gar [13]. These findings indicate that the intron-containing *rhodopsin* gene changed its distribution pattern to exclusive and abundant expression in the pineal gland after branching of the Holostei. Thus, to compare the distribution patterns of the intron-less and intron-containing *rhodopsin* genes of teleost fishes in the Osteoglossomorpha and Elopomorpha, we conducted in situ hybridization analysis in the retina and pineal gland of these fishes (Figs. 2, 3, 4). In the retina of Australian bonytongue (*S. jardinii*), which belongs to the same genus as Asian arowana in the Osteoglossomorpha, we observed the expression of mRNA of the intron-less *rhodopsin* gene in the outer nuclear layer (Figs. 2A, C). However, we could not detect the expression signals of the intron-containing *rhodopsin* gene in the retina (Figs. 2B, D). By contrast, the Australian bonytongue pineal gland abundantly expressed mRNA of the intron-containing *rhodopsin* gene, but not mRNA of the intron-less *rhodopsin* gene (Figs. 2E–H). In addition, analysis of the *rhodopsin* genes in Japanese eel in the Elopomorpha revealed that the photoreceptor cells in the retina expressed mRNAs of two intron-less *rhodopsin* genes (*fw-rho* and *ds-rho*), but not mRNA of the intron-containing *rhodopsin* gene (Figs. 3A–F). We also observed that the Japanese eel pineal gland expressed mRNA of the intron-containing *rhodopsin* gene, but not mRNAs of the intron-less *rhodopsin* genes (Figs. 3G–L).

Among the fishes in the Teleostei we investigated, Atlantic tarpon and Indo-Pacific tarpon in the Elopomorpha are the only species that have the *pinopsin* gene in their genomes. Thus, we analyzed the distribution patterns of transcripts of *rhodopsin* and *pinopsin* genes in the retina and pineal gland of Atlantic tarpon. The expression signals of the intron-less *rhodopsin* gene were strongly detected in the photoreceptor cells of the retina, but not in the pineal gland (Figs. 4A, D, G and J). By contrast, the transcript of the intron-containing *rhodopsin* gene was abundantly expressed in the pineal gland, but not in the retina (Figs. 4B, E, H and K). In addition, we observed that the transcript of the *pinopsin* gene was also expressed in the pineal gland, but not in the retina (Figs. 4C, F, I and L). These findings in Australian bonytongue, Japanese eel and Atlantic tarpon indicate the common expression patterns of *rhodopsin* genes in these teleost fishes, namely expression of the intron-less *rhodopsin* gene in the retina and expression of the intron-containing *rhodopsin* gene in the pineal gland. This expression pattern of the intron-containing *rhodopsin* gene is different from that in spotted gar [13]. This comparison suggests that the intron-containing *rhodopsin* gene changed its expression pattern to one restricted to the pineal gland in the common ancestor of the Teleostei. On the other hand, the abundant expression of the *pinopsin* gene in the pineal gland is conserved between non-teleost fishes (spotted gar, Siberian sturgeon and gray bichir) and a teleost fish (Atlantic tarpon).

Next, we compared the expression patterns of the intron-containing *rhodopsin* gene and the *pinopsin* gene on the Atlantic tarpon pineal gland. We conducted in situ hybridization analysis in the sagittal sections of the Atlantic tarpon pineal gland (Figs. 4M–P). Our analysis showed that the expression signals of the intron-containing *rhodopsin* gene were broadly detected from the rostral to caudal region but were weaker in the caudal region. By contrast, the expression signals of the *pinopsin* gene were restricted to the caudal region. This shows that the regions where the intron-containing *rhodopsin* gene and the *pinopsin* gene mainly function are different in the Atlantic tarpon pineal gland.

In the analysis of the expression patterns of the *rhodopsin* and *pinopsin* genes in Atlantic tarpon, we noticed an anatomical characteristic of the cranium. From the dorsal side of Atlantic tarpon (body length: ~ 10 cm), we could observe a small dusky-red spot in the center of the brain without removing the skin and cranium (Fig. S1A). To analyze the morphology of the cranium, we conducted Alcian Blue staining on sagittal sections of the head including the cranium (Fig. S1B). A pit-like structure of the chondrocranium was formed under the bony cranium and corresponded to the dusky-red spot observed from the dorsal side. Moreover, the pineal gland was located under this pit-like structure. This cranium structure is similar to those of lampreys and cartilaginous fishes [26]. However, this structure is not common in the Teleostei and has only been reported in several species such as rainbow trout (*Oncorhynchus mykiss*) and gilthead seabream (*Sparus aurata*) [27–29]. This morphological characteristic of the cranium is considered to contribute to the improvement of light transmission to the pineal gland in Atlantic tarpon.

### Spectral analysis of rhodopsin and pinopsin proteins

**Fig. 5:**
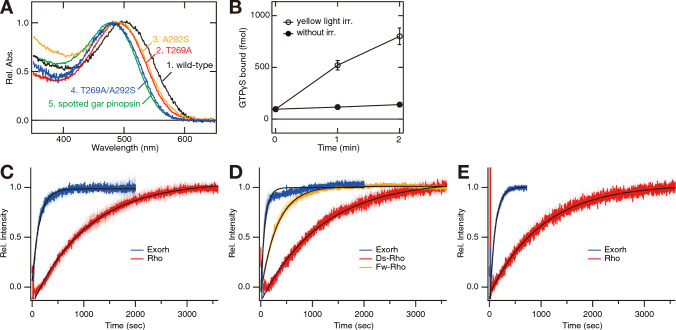
Molecular properties of rhodopsin and pinopsin proteins from fishes in the Actinopterygii. **A,** Comparison of the absorption spectra of wild-type and mutants of Atlantic tarpon pinopsin. Absorption spectra of wild-type (curve 1, λmax = 499 nm), T269A mutant (curve 2, 489 nm), A292S mutant (curve 3, 490 nm) and T269A/A292S mutant (curve 4, 481 nm) were normalized to be ~ 1.0 at λmax. Absorption spectrum of spotted gar pinopsin (curve 5, 478 nm) is also shown. **B**, Activation of transducin by Atlantic tarpon pinopsin protein. The transducin activation ability was measured using the GTPγS binding assay in the dark (closed circle) and after yellow light (> 500 nm) irradiation (open circles). Data were obtained at 15 ºC and are presented as the means ± S.E.M of three independent experiments. **C**, Comparison of the decay of meta II of rhodopsin proteins encoded by the intron-containing gene (Exorh, blue trace) and intron-less gene (Rho, red trace) of Australian bonytongue. **D**, Comparison of the decay of meta II of rhodopsin proteins encoded by the intron-containing gene (Exorh, blue trace) and intron-less genes, *fw-rho* (Fw-Rho, orange trace) and *ds-rho* (Ds-Rho, red trace), of Japanese eel. **E**, Comparison of the decay of meta II of rhodopsin proteins encoded by the intron-containing gene (Exorh, blue trace) and intron-less gene (Rho, red trace) of Atlantic tarpon. The traces in **C**–**E** indicate the average calculated based on three independent measurements with standard errors shown by shaded region. The data in **C**–**E** were fitted by a single exponential function to estimate the decay time constant as follows; Australian bonytongue rhodopsin encoded by the intron-less gene, 740 s; Australian bonytongue rhodopsin encoded by the intron-containing gene, 98 s; Japanese eel rhodopsin encoded by the intron-less gene *fw-rho*, 223 s; Japanese eel rhodopsin encoded by the intron-less gene *ds-rho*, 817 s; Japanese eel rhodopsin encoded by the intron-containing gene, 56 s; Atlantic tarpon rhodopsin encoded by the intron-less gene, 709 s; Atlantic tarpon rhodopsin encoded by the intron-containing gene, 70 s

Next, we analyzed the molecular properties of rhodopsin and pinopsin proteins of Australian bonytongue, Japanese eel and Atlantic tarpon. We prepared the recombinant proteins of rhodopsin and pinopsin in cultured cells and purified the photo-pigments after the addition of 11-*cis* retinal (Fig. S2). All of the rhodopsin proteins encoded by the intron-less *rhodopsin* genes and the exo-rhodopsin proteins encoded by the intron-containing *rhodopsin* genes had λmax at around 500 nm, with the exception of Japanese eel deep-sea type rhodopsin protein. The λmax of Japanese eel deep-sea type rhodopsin protein was located at 483 nm and this blue-shift was previously predicted by the mutations at positions 83 and 292 (D83N/A292S) (in the bovine rhodopsin numbering system) [30, 31]. In addition, the analysis of the Atlantic tarpon pinopsin protein unveiled an interesting spectral shift. Atlantic tarpon pinopsin protein had λmax at 499 nm, which is comparable with that of Atlantic tarpon exo-rhodopsin protein (Figs. S2G, H). The previous reports showed that pinopsin proteins are blue-sensitive opsins with λmax located at 465 ~ 480 nm [16, 17]. Thus, Atlantic tarpon uniquely has green-sensitive pinopsin protein in the pineal gland. We searched for the amino acid residue(s) responsible for this red-shift of Atlantic tarpon pinopsin protein by comparing the amino acid sequences among blue-sensitive *Xenopus tropicalis* and spotted gar pinopsin proteins and green-sensitive Atlantic tarpon pinopsin protein and found that Xenopus and spotted gar pinopsin proteins have Ala269 and Ser292, whereas Atlantic tarpon and Indo-Pacific tarpon pinopsin proteins have Thr269 and Ala292 (Fig. S3). We then performed mutational analysis at positions 269 and 292 of Atlantic tarpon pinopsin protein. The λmax of T269A and A292S single mutant proteins were blue-shifted (~ 10 nm) compared to λmax of the wild-type protein. In addition, T269A/A292S double mutant protein showed a further blue-shift (18 nm) of λmax (481 nm), which is in close agreement with λmax of spotted gar pinopsin protein (478 nm) (Fig. 5A). It is well known that the residues at positions 269 and 292 are responsible for the spectral tuning in vertebrate red-sensitive cone visual pigments and, especially, the mutation at position 269 (alanine or threonine) contributes to the spectral difference between human red- and green-sensitive cone visual pigments [32, 33]. These results indicate that Atlantic tarpon acquired the green-sensitive pinopsin protein through a molecular mechanism which is common with that underlying vertebrate color vision. In addition, we performed G protein activation analysis of Atlantic tarpon pinopsin protein. Previous studies reported that the pinopsin proteins from several species activate transducin in a light-dependent manner [16, 17]. Our analysis also confirmed the coupling of the green-sensitive Atlantic tarpon pinopsin protein with transducin after light irradiation (Fig. 5B). This means that Atlantic tarpon pinopsin protein changed its absorption spectrum without losing its G protein coupling function.

### Analysis of *meta* II of rhodopsin and exo-rhodopsin proteins

Next, we compared the molecular properties of meta II of rhodopsin and exo-rhodopsin proteins. It has been reported that meta II of exo-rhodopsin protein in the pineal gland decays faster than that of the rhodopsin protein in rod cells of the retina [11, 12]. However, our previous study showed that the intron-containing and intron-less *rhodopsin* genes of non-teleost fishes in the Actinopterygii work exclusively in rod cells of the retina and meta II of rhodopsin proteins encoded by these genes decays slowly [13]. These results suggest that the short lifetime of meta II of exo-rhodopsin protein was acquired during the early evolutionary process of the Teleostei, and can contribute to the optimization of the exo-rhodopsin protein for the pineal photoreception under bright conditions by facilitating bleach recovery of photo-pigment. Thus, in this study, we compared the lifetime of meta II among rhodopsin and exo-rhodopsin proteins of teleost fishes in the Osteoglossomorpha and Elopomorpha. To estimate the lifetime of meta II, we measured the release of the retinal from meta II by monitoring the intrinsic tryptophan fluorescence emission. Our analysis showed that meta II of exo-rhodopsin proteins found in the pineal gland of Australian bonytongue, Japanese eel and Atlantic tarpon decays faster than that of rhodopsin proteins in the retina of the teleost fishes (Figs. 5C–E). These results indicate that exo-rhodopsin proteins of teleost fishes in the Osteoglossomorpha and Elopomorpha have meta II with a lifetime shorter than that of rhodopsin proteins of these fishes.

Finally, we searched for the amino acid residue(s) whose mutation(s) results in the short lifetime of meta II of exo-rhodopsin. We compared the sequences of rhodopsin and exo-rhodopsin proteins in the Actinopterygii and picked up nine candidate residues (positions 88, 98, 107, 151, 194, 201, 210, 224, and 277) (Fig. S4). We replaced each residue of Atlantic tarpon intron-containing *rhodopsin* (*exo-rhodopsin*) gene with the corresponding residue encoded by the gray bichir intron-containing *rhodopsin* gene (V88F, A98S, V107E, K151N, P194L, T201E, L210V, S224G, and A277T) and prepared these mutant proteins of Atlantic tarpon exo-rhodopsin after reconstitution with 11-*cis* retinal. All of the mutant proteins had the λmax at around 500 nm (Fig. S5 inset). We measured the decay rate of meta II of these mutant proteins (Fig. S5). Among them, the P194L mutant protein showed a slightly prolonged lifetime of meta II (Fig. S5E), which is consistent with a previous report about rhodopsin proteins of deep-diving vertebrates [34]. However, the other mutant proteins maintained a short lifetime of meta II. Thus, we speculate that not a single mutation but the combination of several mutations contributes to shortening the lifetime of meta II of exo-rhodopsin protein.

## Discussion

Vertebrate genomes have various kinds of opsin genes, most of which have multiple introns in their coding regions [5]. However, the genomes of teleost fishes have an intron-less *rhodopsin* gene together with an intron-containing *rhodopsin* gene, an orthologue of the *rhodopsin* gene found in cyclostomes, cartilaginous fishes, sarcopterygian fishes and tetrapods. Therefore, it is speculated that a unique gene duplication, retroduplication, triggered the formation of two types of *rhodopsin* genes in the Actinopterygii. In this study, to complete an evolutionary scenario for the functional differentiation process of these two *rhodopsin* genes, we analyzed the *rhodopsin* genes of teleost fishes in the Osteoglossomorpha and Elopomorpha, which branched before the diversification in the Clupeocephala, and compared them with those of non-teleost fishes in the Actinopterygii. Our analysis of the synteny blocks of the genes, the distribution pattern of the transcripts from the genes and the molecular property of the proteins that the genes encode supports the possibility that, after branching of the Polypteriformes, the intron-less *rhodopsin* gene emerged by retroduplication and, in the common ancestor of the Teleostei, the parental intron-containing *rhodopsin* gene was specialized for pineal photoreception by changes of the distribution pattern of the transcript and the molecular property of the protein. This stepwise evolutionary model has led to the functional differentiation of two *rhodopsin* genes in the Teleostei.

In this study, we also analyzed other pineal opsin gene, *pinopsin*, in the Actinopterygii. Our previous studies identified the *pinopsin* gene in a wide range of non-teleost fishes including coral catshark (*Atelomycterus marmoratus*), gray bichir, Siberian sturgeon, spotted gar and spotted African lungfish (*Protopterus dolloi*) [13, 17]. Here we isolated the *pinopsin* gene from the genomes of Atlantic tarpon and Indo-Pacific tarpon in the Elopiformes of the Elopomorpha. The *pinopsin* gene is located in the conserved synteny block among non-teleost fishes (gray bichir, sterlet and spotted gar) and a teleost fish (Atlantic tarpon) and is missing in other teleost fishes. This suggests that the *pinopsin* gene was not lost in the common ancestor of the Teleostei but was independently lost in several lineages, such as the Osteoglossomorpha, the Albuliformes, the Anguilliformes and the Clupeocephala, of the Teleostei. According to this model, it is speculated that the common ancestor of the Teleostei maintained the intron-containing and intron-less *rhodopsin* genes and the *pinopsin* gene. The analysis of the expression pattern of the intron-containing *rhodopsin* gene and the *pinopsin* gene in Atlantic tarpon showed that both genes are expressed in the pineal gland, that is, we detected broad expression signals, although weaker signals in the caudal region, of the intron-containing *rhodopsin* gene and restricted expression signals of the *pinopsin* gene in the caudal region. On the other hand, it has been reported that the expression signals of the intron-containing *rhodopsin* gene are broadly detected from the rostral to caudal region of the zebrafish pineal gland [10, 25]. Thus, the expression region of the intron-containing *rhodopsin* gene in zebrafish appears to include both those of the intron-containing *rhodopsin* gene and the *pinopsin* gene in Atlantic tarpon. It should be noted that the Atlantic tarpon pinopsin protein exceptionally forms a green-sensitive opsin whose λmax is comparable with that of the exo-rhodopsin protein encoded by the intron-containing *rhodopsin* gene and maintains the transducin activation ability. This spectral shift of the pinopsin protein resulted in spectral sensitivity that mimics that of the exo-rhodopsin protein, which may have led to the survival of the *pinopsin* gene in tarpon. Therefore, Atlantic tarpon has two types of *rhodopsin* genes and the *pinopsin* gene, like a putative common ancestor in the Teleostei, and remains in an evolutionary intermediate state between non-teleost fishes, whose pineal gland expresses the *pinopsin* gene, and teleost fishes, whose pineal gland expresses the intron-containing *rhodopsin* gene. In addition, it is speculated that the intron-containing *rhodopsin* gene has come to be utilized as a pineal opsin instead of the original pineal opsin, *pinopsin*, in other teleost fishes. We previously proposed an acquisition model for the *pinopsin* gene in the ancestor of vertebrates, namely, that the *pinopsin* gene originally functioned for visual and pineal photoreception and subsequently became specialized for pineal photoreception as a consequence of the acquisition of the *rhodopsin* gene for visual photoreception [17]. Thus, the *pinopsin* gene may have been functionally replaced by the *rhodopsin* gene twice, in the early evolutionary processes of vertebrates and teleost fishes.

**Fig. 6:**
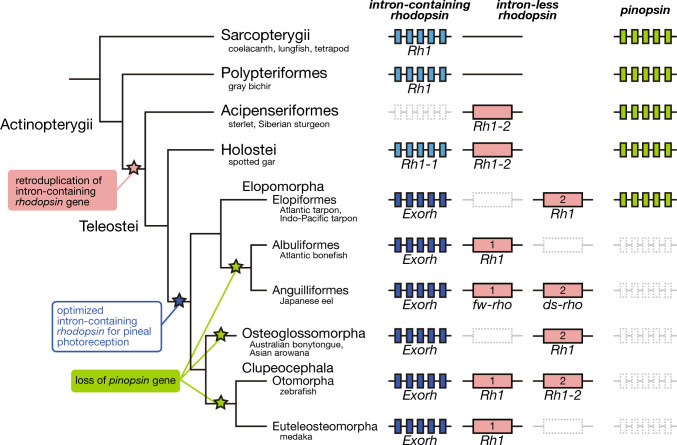
Functional diversification model of *rhodopsin* and *pinopsin* genes in the Actinopterygii. The common ancestor of the Actinopterygii had the intron-containing *rhodopsin* gene for visual photoreception and the *pinopsin* gene for pineal photoreception. After branching of the Polypteriformes, retroduplication of the intron-containing *rhodopsin* gene produced an intron-less *rhodopsin* gene which was utilized for visual photoreception. Among non-teleost fishes, spotted gar in the Holostei has the intron-containing and intron-less *rhodopsin* genes for visual photoreception and the *pinopsin* gene for pineal photoreception. By contrast, in the common ancestor of the Teleostei, the use of the parental intron-containing *rhodopsin* gene changed to utilization for pineal photoreception. Among the teleost fishes we investigated, Atlantic tarpon in the Elopiformes has the intron-less *rhodopsin* gene for visual photoreception and the intron-containing *rhodopsin* gene and the *pinopsin* gene for pineal photoreception. However, the *pinopsin* gene is missing in other teleost fishes, as it has been independently lost in most lineages of the Teleostei

In conclusion, our analysis of the *rhodopsin* and *pinopsin* genes of non-teleost and teleost fishes in the Actinopterygii can provide a complete evolutionary scenario of the differentiation process of these opsin genes in the Actinopterygii, as shown in Fig. 6. The common ancestor of the Actinopterygii would have utilized the intron-containing *rhodopsin* gene for visual photoreception and the *pinopsin* gene for pineal photoreception. After branching of the Polypteriformes, retroduplication of the intron-containing *rhodopsin* gene produced an intron-less *rhodopsin* gene which was exclusively expressed in the retina. Afterward, in the common ancestor of the Teleostei, abundant expression of the newly acquired intron-less *rhodopsin* gene in the retina led to the change of the expression region of the parental intron-containing *rhodopsin* gene from the retina to the pineal gland and the optimization of the molecular property of the protein that the gene encoded for pineal photoreception. Subsequently, in most lineages, except for the Elopiformes, of the Teleostei, the participation of the intron-containing *rhodopsin* gene in pineal photoreception resulted in a loss of the original pineal opsin gene, *pinopsin*. We speculate that unique retroduplication would have caused a “domino effect” on the functional diversification of the visual and pineal opsin in the Actinopterygii. This functional diversification of the *rhodopsin* and *pinopsin* genes was possibly maintained as a result of its contribution to the adaptation to light environments in the habitats of ancestral fishes. Further analysis of the detailed physiological functions of the *exo-rhodopsin* and *pinopsin* genes in non-teleost and teleost fishes will help to answer the question of how light environmental factors contributed to the maintenance of the functional divergence of the opsin genes.

## Supplementary Information

Below is the link to the electronic supplementary material.

Supplementary file1 (PDF 542 KB)

Supplementary file2 (XLSX 18 KB)

## Declarations

### Data availability

All data are available in the main text or the supplementary materials. The sequences reported in this paper have been deposited in the GenBank database (accession numbers: LC818093 ~ LC818097).
