# Supplementary material for: Functional diversification process of opsin genes for teleost visual and pineal photoreceptions
Source: Cell Mol Life Sci. 2024 Oct 8;81(1):428. doi: 10.1007/s00018-024-05461-3 (PMC11461388; doi:10.1007/s00018-024-05461-3)
Supplement: Supplementary file 1 — Supplementary file1 (PDF 542 KB) [file 18_2024_5461_MOESM1_ESM.pdf]

Supporting information for

Functional diversification process of opsin genes for teleost visual and pineal photoreceptions

Authors:

Chihiro Fujiyabu[1], Fuki Gyoja[2], Keita Sato[3], Emi Kawano-Yamashita[4], Hideyo Ohuchi[3], Takehiro G. Kusakabe[2], Takahiro Yamashita[1*]

Affiliations:

[1]Department of Biophysics, Graduate School of Science, Kyoto University, Kyoto 606-8502, Japan; [2]Institute for Integrative Neurobiology and Department of Biology, Graduate School of Natural Science, Konan University, Hyogo 658-8501, Japan. [3]Department of Cytology and Histology, Okayama University Faculty of Medicine, Dentistry and Pharmaceutical Sciences, Okayama 700-8558, Japan; [4]Department of Chemistry, Biology and Environmental Science, Faculty of Science, Nara Women's University, Nara 630-8506, Japan.

*Corresponding Author:

Takahiro Yamashita, Department of Biophysics, Graduate School of Science, Kyoto University, Kyoto 606-8502, Japan.

Email: yamashita.takahiro.4z@kyoto-u.ac.jp

Supplementary Figure 1

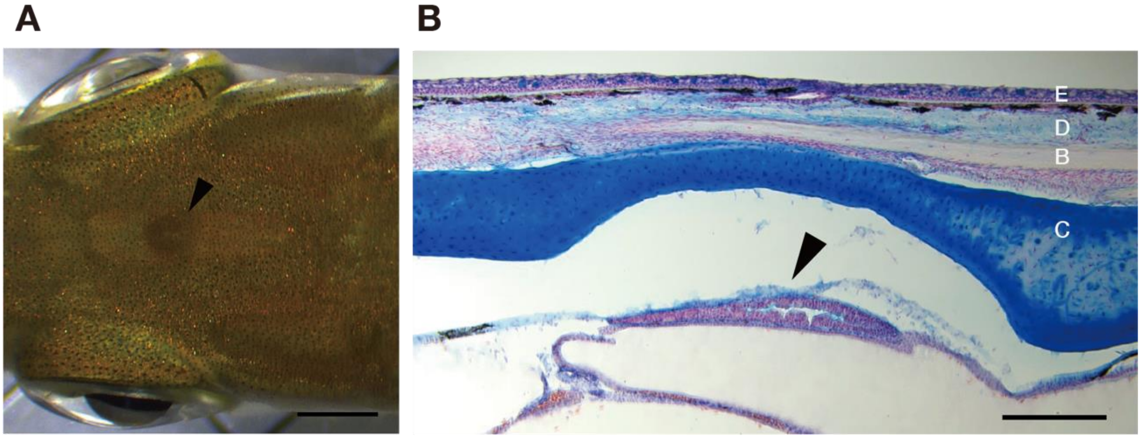

Supplementary Figure 1 Anatomical characteristics of the Atlantic tarpon cranium

A, Atlantic tarpon head viewed from the dorsal side. Rostral is to the left. A small dusky-red spot (indicated by arrowhead) is observed in the center of the brain without removing the skin and cranium. Scale bar: 2 mm. **B**, Sagittal section of Atlantic tarpon head including the cranium around the pineal gland (indicated by arrowhead) with Alcian Blue staining. Rostral is to the left, and dorsal is up. Scale bar: 200 µm. Abbreviations: E, epidermis; D, dermis; B, bony cranium; C, chondrocranium.

Supplementary Figure 2

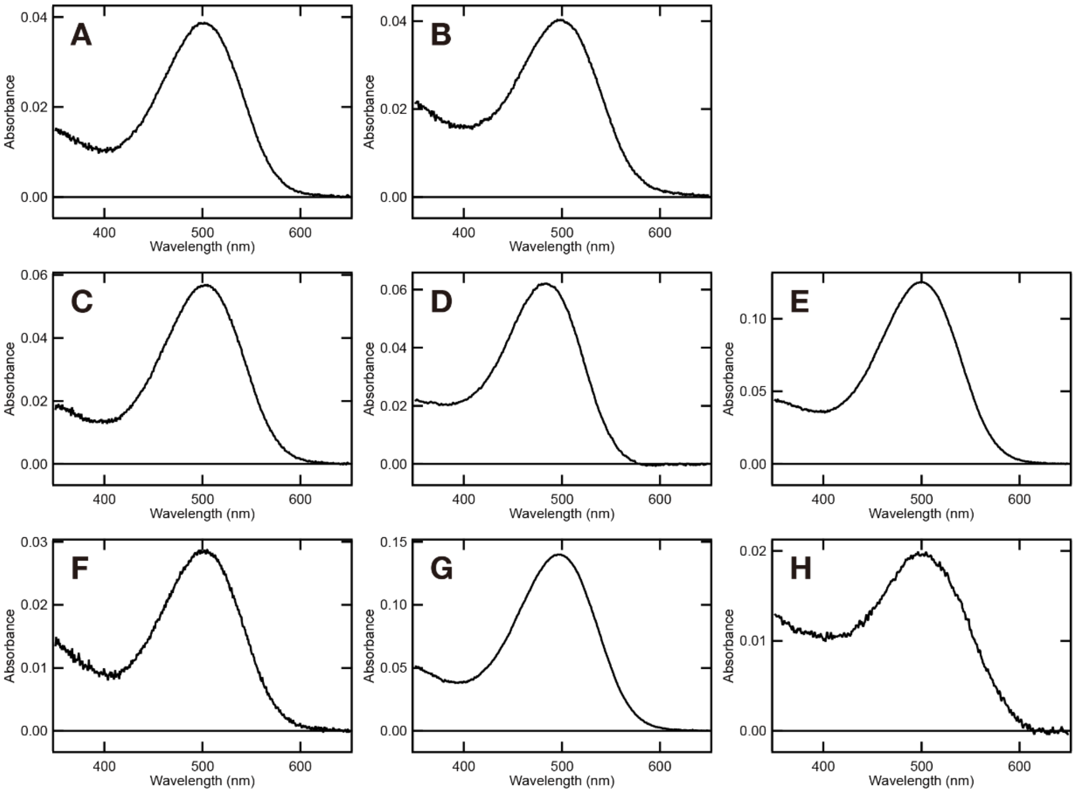

Supplementary Figure 2 Absorption spectra of rhodopsin and pinopsin proteins of the basal Teleostei

The absorption spectra of Australian bonytongue rhodopsin proteins encoded by the intron-less *rhodopsin* gene (**A**, λmax = 501 nm) and the intron-containing *rhodopsin* gene (**B**, 498 nm), Japanese eel rhodopsin proteins encoded by the intron-less *rhodopsin* genes, *fw-rho* (**C**, 502 nm) and *ds-rho* (**D**, 483 nm), and the intron-containing *rhodopsin* gene (**E**, 499 nm), Atlantic tarpon rhodopsin proteins encoded by the intron-less *rhodopsin* gene (**F**, 501 nm) and the intron-containing *rhodopsin* gene (**G**, 496 nm), and Atlantic tarpon pinopsin protein encoded by the *pinopsin* gene (**H**, 499 nm) are shown.

Supplementary Figure 3

```
                               260                     280
                                |....|.... |....|.... |....|.... |....|....
bovine Rhodopsin               AFLICWLPYA GVAFYIFTHQ GSDFGPIFMT IPAFFAKTSA
Atlantic tarpon Pinopsin       AFLICWCPYT TFALVVAIDK DIKISPTLAS MPAYFSKTAT
Indo-Pacific tarpon Pinopsin   AFLICWCPYT TFALVVAIDK DIKISPTLAS MPAYFSKTAT
spotted gar Pinopsin           AFLVCWLPYA TFAMVVAIDK NIVIQPTLAS MPSYFSKTAT
xenopus tropicalis Pinopsin    AFLICWLPYA SFAVVVAVNK DVVIEPTVAS LPSYFSKTAT
```

Supplementary Figure 3 Comparison of the amino acid sequences among pinopsin proteins

Amino acid sequences of bovine rhodopsin (K00506), Atlantic tarpon pinopsin (LC818095), Indo-Pacific tarpon pinopsin (XM_036523016), spotted gar pinopsin (XM_015367820), and *X. tropicalis* pinopsin (XM_002934345) were aligned using ClustalW. The numbers above the sequence alignment indicate the residue positions of bovine rhodopsin. Two residues at positions 269 and 292 are highlighted in red.

Supplementary Figure 4

```
                                     70         80         90        100        110        120
                             ....|....| ....|....| ....|....| ....|....| ....|....| ....|....|
bovine Rh1                   VTVQHKKLRT PLNYILLNLA VADLFMVFGG FTTTLYTSLH GYFVFGPTGC NLEGFFATLG
gray bichir Rh1              VTVQHKKLRS PLNYILLNLA VSGLFMVFGG FTTTLYTSMH GYFIFGETGC NLEGFFATLG
Siberian sturgeon Rh1-2      VTIQHKKLRT PLNYILLNLA VADLFMVFGG FTTTMYTSMN GYFVFGTTGC NIEGFFATLG
spotted gar Rh1-1            VTIQHKKLRT PLNYILLNLA VADLFMITGG FTTTMYTSMH GYFVFGPAGC NVEGFFATLG
spotted gar Rh1-2            VTIEHKKLRT PLNYILLNLA VGDLFMVFGG FTTTMYTSMN GYFVFGTTGC NIEGFFATLG
Australian bonytongue Rho    VTIEHKKLRT PLNYILLNLA VADLFMVFGG FTTTMYTSMN GYFVFGTTGC NIEGFFATTG
Japanese eel fw-rho          VTIEHKKLRT PLNYILLNLA VADLFMVFGG FTTTMYTSMH GYFVFGPTGC NIEGFFATLG
Japanese eel ds-rho          VTIEHKKLRT PLNYILLNLA VANLFMVFGG FTTTMYTSMH GYFVFGETGC NLEGYFATLG
Atlantic tarpon Rho          VTIEHKKLRT PLNYILLNLA VADLFMVFGG FTTTMYTSMN GYFVFGETGC NIEGYFATLG
Australian bonytongue Exorh  VTIQHKKLRT PLNYILLNLA VADLFMVIGG FTTTLYTALH GYFILGVLGC NIEGFFATLG
Japanese eel Exorh           VTVQHKKLRT PLNYVLLNLA VADLFMVVGG FTTTLYTALH GYFALGIMGC NIEGFFATLG
Atlantic tarpon Exorh        VTIQHKKLRT ALNYILLNLA VADLFMVVGG FTTTLYTALH GYFVLGVMGC NIEGFFATMG

                                    130        140        150        160        170        180
                             ....|....| ....|....| ....|....| ....|....| ....|....| ....|....|
bovine Rh1                   GEIALWSLVV LAIERYVVVC KPMSNFRFGE NHAIMGVAFT WVMALACAAP PLVGWSRYIP
gray bichir Rh1              GEIGLWSLVV LAIERYIVVC KPMANFRFGE NHAIMGVVFT WIMALSCAAP PLLGWSRYIP
Siberian sturgeon Rh1-2      GEIGLWCLVV LAIERYIVVC KPMSNFRFGE NHAIMGVVFT WIMALSCAAP PLFGWSRYIP
spotted gar Rh1-1            GEIALWSLVV LAIERYIVVC KPVSNFRFGE NHAVMGVALT WIMALACAAP PLFGWSRYIP
spotted gar Rh1-2            GMISLWSLVV LAVERYLVVC KPMSNFRFGE SHSIMGVLFT WVMACACAVP PLFGWSRYIP
Australian bonytongue Rho    GEIALWSLVV LAIERYVVVC KPMSNFRFGE NHAIMGVAFT WVMACSCSVP PLFGWSRYIP
Japanese eel fw-rho          GEIALWCLVV LAIERWVVVC KPMSNFRFGE NHAIMGVAFT WLMALACAAP PLFGWSRYIP
Japanese eel ds-rho          GEISLWSLVV LAIERWVVVC KPMSNFRFGE NHAIMGLAFT WIMANTCALP PLFGWSRYIP
Atlantic tarpon Rho          GEIGLWSLVV LAIERYVVVC KPMSNFRFGE NHAIMGVAFT WIMANACAAP PLFGWSRYIP
Australian bonytongue Exorh  GEIALWSLVV LAVERYIVVC KPVSIFRFGE THAIIGVALT WVMSLTCAVP PLLGWSRYIP
Japanese eel Exorh           GEIALWSLVV LAIERYIVVC KPVSSFRFGE KHAILGVALT WIMALTCAVP PLLGWSRYIP
Atlantic tarpon Exorh        GEIGLWSLVV LAIERYIVVC KPMTNFRFGE KHAIMGVALT WVMALSCAVP PLLGWSRYIP

                                    190        200        210        220        230        240
                             ....|....| ....|....| ....|....| ....|....| ....|....| ....|....|
bovine Rh1                   EGMQCSCGID YYTPHEETNN ESFVIYMFVV HFIIPLIVIF FCYGQLVFTV KEAAAQQQES
gray bichir Rh1              EGMQCSCGID YYTLKPEVNN ESFVIYMFIV HFTIPMIVIF FCYGRLVCTV KEAAAQQQES
Siberian sturgeon Rh1-2      EGMQCSCGID YYTLKPEVNN ESFVIYMFVV HFFIPLIIIS FCYGRLVCTV KEAAAQQQES
spotted gar Rh1-1            EGMQCSCGID YYTVTPEVNN KSFVIYMFIV HFSIPLTVIF FCYGRLVCTV KQAASQQQES
spotted gar Rh1-2            EGMQCSCGID YYTLKPEVNN ESFVIYMFTV HLLLPFSVVF FCYGRLVCTV KAAAAQQQES
Australian bonytongue Rho    EGMQCSCGID YYTMKPEVNN YSFVIYMFIV HFSIPLTIVF FCYGRLVCTV KEAAAQQQES
Japanese eel fw-rho          EGMQCSCGID YYTPNPETYN ESFVIYMFVC HFTIPLTVVS FCYGRLVCTV KEAAAQQQES
Japanese eel ds-rho          EGMQCSCGVD YYTLKPEVNN ESFVIYMFIV HFSIPLTIIS FCYGRLVCTV KEAAAQQQES
Atlantic tarpon Rho          EGMQCSCGID YYTLKPEVNN ESFVIYMFIV HFTIPLTIVF FCYGRLVCTV KEAAAQQQES
Australian bonytongue Exorh  EGMQCSCGID YYTPKPEINN TSFVIYMFIL HFTIPLVIIF FCYSRLLCTV RAAAAQQQES
Japanese eel Exorh           EGMQCSCGID YYTPKPELNN VSFVIYMFVL HFSIPLVVIF FCYSRLLCTV RAAAAQQQES
Atlantic tarpon Exorh        EGMQCSCGID YYTPKPELNN TSFVIYMFIL HFSIPLLVIF FCYSRLLCTV RAAAAQQQES

                                    250        260        270        280        290        300
                             ....|....| ....|....| ....|....| ....|....| ....|....| ....|....|
bovine Rh1                   ATTQKAEKEV TRMVIIMVIA FLICWLPYAG VAFYIFTHQG SDFGPIFMTI PAFFAKTSAV
gray bichir Rh1              ETTQRAEREV TRMVVVMVVG FLICWVPYAS VAWYIFTHQG TDFGPVFMTA PAFFAKSASL
Siberian sturgeon Rh1-2      ETTQRAEREV TRMVVLMVIS FLVCWTPYAS VAWYIFTHQG SHFGPVFMTT PAFFAKSSAL
spotted gar Rh1-1            ETTQRAEKEV TRMVVVMVIS FLVCWLPYAS VAFYIFVNQG SNFGPVFMTA PAFFAKSSAL
spotted gar Rh1-2            ETTQRAEREV TRMVVLMVIS YIVCWTPYGS VAWYIFTHKG ADFGPVFMTG PAFFAKSSAL
Australian bonytongue Rho    ETTQRAEREV TRMVIIMVIS FLVCWLPYAG VAWYIFTHQG SDFGPVFMTA PAFFAKAASI
Japanese eel fw-rho          ETTQRAEREV TRMVVIMVIS FLVCWVPYAS VAWYIFTHQG SSFGPIFMTI PAFFAKSSAL
Japanese eel ds-rho          ETTQRAEREV TRMVVIMVIA FLVCWIPYAS VAWYIFTHQG STFGPVFMTV PSFFAKSSAI
Atlantic tarpon Rho          ETTQRAEREV TRMVIIMVLS FLVCWIPYAS VAWYIFTHQG SSFGPVFMTA PAFFAKSSAL
Australian bonytongue Exorh  ETTQRAEKEV TRMVVVMVIA FLICWLPYAS VAWYIFANQG VDFGPVFMTV PAFFAKSAAL
Japanese eel Exorh           ETTQRAEKEV TRMVVVMVFS FLVCWVPYAS VAWYIFANQG ADFGPVFMTI PAFFAKSAAL
Atlantic tarpon Exorh        ETTQRAEKEV TRMVVVMVIS FLVCWLPYAS VAWYIFANQG TDFGPVFMTV PAFFAKSAAL
```

Supplementary Figure 4 Comparison of the amino acid sequences among rhodopsin proteins in the Actinopterygii

Amino acid sequences of bovine rhodopsin encoded by the intron-containing gene (*Rh1*) (K00506), gray bichir rhodopsin encoded by the intron-containing gene (*Rh1*) (LC438460), Siberian sturgeon rhodopsin encoded by the intron-less gene (*Rh1-2*) (LC438462), spotted gar rhodopsin encoded by the intron-

containing gene (*Rh1-1*) (XM_006630940), spotted gar rhodopsin encoded by the intron-less gene (*Rh1-2*) (XM_006630625), Australian bonytongue rhodopsin encoded by the intron-less gene (*Rho*) (LC818096), Japanese eel rhodopsins encoded by the intron-less genes, *fw-rho* (LC464071) and *ds-rho* (LC464070), Atlantic tarpon rhodopsin encoded by the intron-less gene (*Rho*) (LC818093), Australian bonytongue rhodopsin encoded by the intron-containing gene (*Exorh*) (LC818097), Japanese eel rhodopsin encoded by the intron-containing gene (*Exorh*) (LC464064), and Atlantic tarpon rhodopsin encoded by the intron-containing gene (*Exorh*) (LC818094) were aligned using ClustalW. The numbers above the sequence alignment indicate the residue positions of bovine rhodopsin. Residues at positions 88, 98, 107, 151, 194, 201, 210, 224, and 277 are highlighted in red.

Supplementary Figure 5

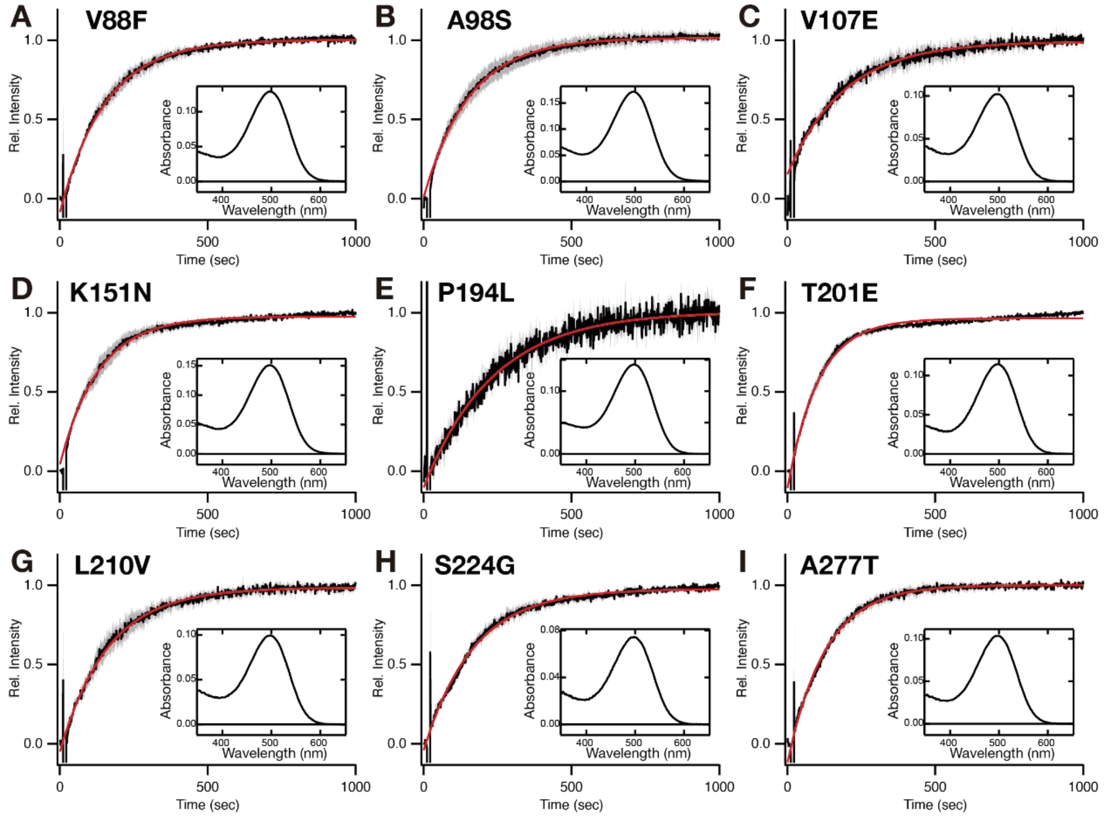

Supplementary Figure 5 Molecular properties of the mutants of Atlantic tarpon exo-rhodopsin protein encoded by the intron-containing *rhodopsin* gene

Comparison of the decay of meta II of exo-rhodopsin mutants, V88F (**A**), A98S (**B**), V107E (**C**), K151N (**D**), P194L (**E**), T201E (**F**), L210V (**G**), S224G (**H**), and A277T (**I**). Black trace in each panel indicates the average calculated based on three (**A**–**C**, **E**, **G**–**I**) or four (**D**, **F**) independent measurements with standard errors shown by shaded region. The data were fitted by a single exponential function (red curve) to estimate the decay time constant as follows: 106 sec for V88F, 107 sec for A98S, 132 sec for V107E, 90 sec for K151N, 163 sec for P194L, 72 sec for T201E, 112 sec for L210V, 115 sec for S224G, and 88 sec

for A277T. (inset) Absorption spectrum of the rhodopsin mutant in each panel. λmax of these proteins was estimated to be 497 nm (**A**, **E**, **F**) or 496 nm (**B**–**D**, **G**–**I**) or, which are comparable with that of wild-type protein (496 nm).
